# The Buffalo Medical and Surgical Journal

**Published:** 1862-07

**Authors:** 


### EDITORIAL DEPARTMENT.

### THE BUFFALO MEDICAL AND SURGICAL JOURNAL.

The present number completes the first volume of our Journal, and we shall be indulged a familiar word with our friends, on an occasion to us, so full of interest. It is proper and desirable that we look again over the ground we have passed, hoping to gain some lessons for future guidance, the more since it will open to us anew our own imperfect efforts. Altogether unaccustomed to the duties and labors of our new position, with constantly increasing demands upon our time and thoughts, we have conducted the pages of the Journal thus far; and we desire most heartily to thank our friends for their numerous kind expressions of interest and favor; for their contributions to our pages; for their prompt remittances; and for the evidences we have received from all directions, that the profession are to be safely relied upon, for the success of our young and unpretending Journal. It has been our desire to furnish our readers an original journal, since we did not presume but most of them read other medical periodicals to so great an extent, that selections would hardly be new or interesting, yet not presuming that our pages might not be profitably occupied, to some extent at least, by extracts from both foreign and domestic Journals. We have hoped, (and in this respect have not been disappointed,) to obtain and preserve the experience and observation, of not only the men of our own time and country, but more especially the men whose field of research is within the limits of our circulation and influence. We have already collected something, which though small in amount, we hope has been true in fact, and correct in principle, and will prove only as the beginning of a record, which shall represent the best, most progressive, most truthful, and at the same time, most practically valuable experience.

Though we have called our Journal, young and unpretending, yet we hope no one will think it unambitious. We call it unpretending because it is small; but we expect it will grow. It was commenced small, that it might grow larger and better, and the experience of its first year fully demonstrates the wisdom of the plan, for medical journals the last year have grown smaller and fewer. There are many reflections suggested by the review of the past, some of which we do not desire to intrude upon our readers. The difficulties and labors of conducting the Journal have been greatly increased by the all-absorbing events occurring in the history of our country. The one all-controlling topic of thought and effort, has in great degree, drawn off attention from the ordinary channels and investigation. Great numbers of the profession have forgotten every professional consideration and interest, and with self-sacrificing heroism devoted themselves to the duties of military practice; thus, for a time, neglecting their ordinary professional researches and observations, and their accustomed contributions. Again this has been very unfavorable for extensive circulation. Medical journals have been discarded as a luxury in which the times would not allow an indulgence. But of this we have no reason to complain; we have only reason for thankfulness and obligation. Our Journal has been extensively circulated; received with many expressions of satisfaction and favor, and paid for with cheerfulness and promptitude. For all this we are much obliged. From brother Editors we have received kindness and cordiality; by the daily press have been very pleasantly and flatteringly noticed, and all our Editorial relations have been exceedingly pleasant. With authors and publishers we have made many and valuable acquaintances, and cannot be too ardent in expressing our obligations for the additions made to our library, through their generosity. Our exchanges must not be omitted in connection with other favors and courtesies. They are in many instances valuable works upon which great labor and outlay are expended, or reprints of foreign journals which we very highly prize.

For our future we shall also be expected to say a word. Our plan of operation must conform for at least our next volume, very much to the style of the first, since we judge it more prudent to "live upon our income, than to borrow." As heretofore we shall prefer soundness to mere novelty, and matter of constant practical value, to the most plausible and ingenious theory, the relations of diseases which are common and constant to those rarely seen, or operations which but few surgeons in the world ever perform. We shall hope in so far as our limits permit, to keep pace, and publish important physiological and pathological observation as it may be offered to the profession, at home or abroad. We are girding ourselves anew for the duties and responsibilities of our next volume, and hope that the experiences of the past, may contribute to our ability to conduct its pages with greater satisfaction to ourselves, and more acceptably to our readers.

We have at heart the benefit of the medical profession, and shall hope to do our humble part in protecting it from foes within, and open enemies without; to encourage and strengthen the kindlier relations of professional intercouse, and to elevate her members, high above the common level of empiricism and charlatanry. The medical profession is with us a sort of household god, and the honest, progressive, and manly practice of her art, the most sublime and God-like mission; when degraded to the low level of a trade, and practiced with cunning tricks, her votaries are fallen, and can no longer "walk the golden streets of truth and life."

With no lengthy apologies for the imperfections of the past, or sanguine assurances for the future, let us again remind our readers and friends, that not ourselves alone are responsible for the issues of this enterprise. It can only be successfully conducted with your hearty co-operation and support.

